# The Modeling of Super Deep Learning Aiming at Knowledge Acquisition in Automatic Driving

**DOI:** 10.1155/2022/8928632

**Published:** 2022-04-09

**Authors:** Yin Liang, Zecang Gu, Zhaoxi Zhang

**Affiliations:** ^1^China University of GeoScience, Wuhan, China; ^2^Apollo Japan Co., Ltd. CTO, Tianjin, Japan; ^3^Shijiazhuang Tiedao University, Shaoxing, China

## Abstract

In this paper, we proposed a new theory of solving the multitarget control problem by introducing a machine learning framework in automatic driving and implementing the acquisition of excellent drivers' knowledge. Nowadays, there still exist some core problems that have not been fully realized by the researchers in automatic driving, such as the optimal way to control the multitarget objective functions of energy saving, safe driving, headway distance control, and comfort driving. It is also challenging to resolve the networks that automatic driving is relied on and to improve the performance of GPU chips on complex driving environments. According to these problems, we developed a new theory to map multitarget objective functions in different spaces into the same one and thus introduced a machine learning framework of SDL (super deep learning) for optimal multitarget control based on knowledge acquisition. We will present in this paper the optimal multitarget control by combining the fuzzy relationship of each multitarget objective function and the implementation of excellent drivers' knowledge acquired by machine learning. Theoretically, the impact of this method will exceed that of the fuzzy control method used in the automatic train.

## 1. Introduction

The automatic driving is one of the most outstanding and promising application areas of artificial intelligence. Olaverri-Monreal [1] suggested that it should be very important to understand the actions of different road drivers and their reactions to the vehicles. Howard et al. [2] proposed a method to generate adversarial self-driving scenarios into the training set of the self-driving policy to obtain safer self-driving behavior by using Bayesian optimization. And however, there are still few concerns on the method of constructing the knowledge acquisition model of excellent drivers and the optimal multitarget control, which are essential for the automatic driving.

The most representative control method in automatic driving is based on networks. The autonomous highway vehicle (AHV) delivered its status and predetermined headings, such as its ID, the time, its location, its orientation, its speed, and the predetermined route, to the servers through networks. This information composed the spatial decisive data to help the network servers directly control the AHV [3, 4]. However, the automatic driving technology of vehicles is far more complex than that of trains. For example, the right moment and the appropriate distance between two driving cars to keep away from each other are much fuzzier than objective data, while the objective data are necessary for the strict machine control in practice. Kalmbach et al. [5] also indicated that the fundamental challenge to optimize the network of automatic driving is the limited knowledge about the future changes in the environment. Furthermore, the objective functions of automatic driving, which not only refer to the pilot process but also involve more issues about safe driving, headway distance control, comfort driving, are separately defined in the different spaces so that it is dramatically difficult to directly control them only based on networks.

Another representative automatic driving method is to build the control system with high-performance chips in order to process the world-scale data online [6]. Nevertheless, since the automatic driving system has to face the complex road and driving conditions and also tries to solve the optimization problems of safe driving, headway distance control, comfort driving simultaneously, the control system equipped with high-performance chips will be incapable of optimizing the multitarget functions based on fuzzy degree.

In order to explore those problems, we build up a framework, so-called super deep learning, to map the multitarget objective functions in different spaces into the same space so that it is possible to optimize multitarget control based on knowledge acquisition.

## 2. Some Barriers Automatic Driving Needs to Breakthrough

In this section, we will have a discussion on some kernel problems in automatic driving about human-machine judgment conflict, human-machine sensory integration, and human-machine authority transition, so as to explore the new theoretical methods and build efficient models for the optimization of the multitarget functions [7]. Badue et al. [8] stated that the architecture of the autonomy system of self-driving cars would be organized into the perception system and the decision-making system. They also had a detailed discussion on the subsystems dealing with similar problems in the paper [7] in their survey.

### 2.1. Human-Machine Judgment Conflict

In Google's automatic driving experiment, when the automatic vehicle met a barrier in front and tried to turn right for the avoidance, a truck came from the right-back, and then, a heavy accident happened because the truck's driver thought that Google's vehicle should stop in front of the barrier.

### 2.2. Human-Machine Sensory Integration

According to the investigation of a company, there are 41% people think the best way is to stay away as far as they can when meeting an automatic vehicle, while others suggest that keeping a fixed or a little near distance would be better. And even more there are a few persons with curiosity who will catch up with the vehicle in front. It is a knotty problem in automatic control to integrate the sensory deviations between machines and human beings, in order to guide the vehicles in the closest way to people's driving.

### 2.3. Human-Machine Authority Transfer

Consciousness of human beings cannot be transferred to machines like the authority transition between them. For instance, it is easy to miss an opportunity of avoiding accidences to happen when machines chose different plans from human beings in emergency during the authority transition.

### 2.4. Trolley Problem

The famous trolley problem is described as how to reduce the number of sacrifices to the lowest in emergency. It is related to not only intricate ethical issues but also technical difficulties so that nobody up to now has proposed a valuable solution to the trolley problem of automatic driving.

### 2.5. Fuzzy Control

People are aware that there is a determined control point that makes the automatic driving different. Therefore, it is impossible for the traditional methods to solve the problems of multitarget functions simultaneously, such as safe driving, headway distance control, comfortable driving, when the speed of the automatic vehicle is changing in the complicated road. The only solution to the multitarget control is to rely on the experience of excellent drivers, which is what the machine learning model is skilled in.

## 3. The Model of Super Deep Learning (SDL)

With the rapid development of the machine learning model in this climax of AI, the deep learning technology will be the fundamental to solve probabilistic problems. Cavalcante et al. [9] presented a visual global localization system based on deep neural networks to locate self-driving cars accurately. The large-scale applications of deep learning will be carried out to affect the variant aspects of society rather than any other technology in the past.

**Figure 1:**
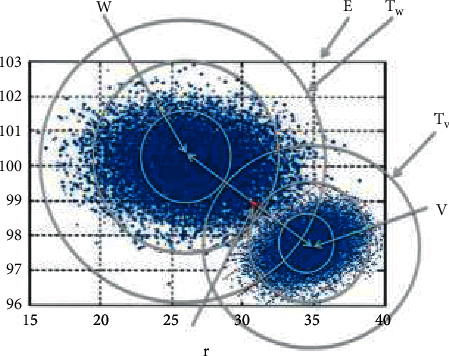
The best classification for probability space.

Figure 1 is the schematic diagram of the best classification for probability space. As shown in Figure 1, the main problem, which AI need to solve nowadays, is to get the probability distribution of the objective function through machine learning and then decide which probability distribution is closest to a point in the space that is solving the problem of best classification of probability space in the Euclidean space.

In Figure 1, assuming there are two centers, *w* and *v*, which belong to the different probability spaces composed by the probability distribution W and V in the Euclidean space *E*, there is a point *r*, and the major task of machine learning is to figure out which is closest to *r*, *w*, or *v*. This proposition is a typical pattern recognition problem and can be easily solved by working out the rigorous distance formula between *w* and *v*. As the kernel problem in machine learning, the distance between probability distributions is getting more and more attention from many AI researchers [10–16].

Based on the theory proposed by the Russian mathematician Andrey Kolmogorov, “the probability space is measurable space with the measure of 1,” lemma 1 can be proposed as “there is only one probability distribution in the probability space, and the distance in probability space is a way to solve the distance between the Euclidean space and the probability space.” Data used by AI are located in one of the probability spaces in the Euclidean space, so the classification criteria in AI should be dependent on the distance leaping over the Euclidean space to the probability space.

Based on lemma 1 mentioned above, lemma 2 can be introduced as the distance, whose probability distribution is 1 in the probability space, is zero.

The formula about the distance leaping over the Euclidean space to the probability space can be developed according to lemma 2. Assuming there two points, *w*_*j*_ (*j* = 1, 2,…, *n*) belonging to the vector *W* is the center of the probability distribution *T*_*w*_, and *v*_*j*_ (*j* = 1, 2,…, *n*) belonging to the vector *V* is the center of the probability distribution *T*_*v*_, and the distance between *v*_*j*_ and *w*_*j*_ leaping over the Euclidean space and the probability space is as follows:

(1) $$\begin{array}{c} G\left(W,V\right)={\left\{\overset{n}{\underset{j=1}{\sum }}{\left(w_{j}-v_{j}\right)}^{2}\right\}}^{1/2}, \end{array}$$

(2) $$\begin{array}{c} \left(w_{j}-v_{j}\right)=\left\{\begin{array}{cc} 0 & \left|w_{j}-v_{j}\right|\leq \Delta_{j}^{\left(vj\right)}\\ \left|w_{j}-v_{j}\right|-\Delta_{j}^{\left(vj\right)} & \left|w_{j}-v_{j}\right|>\Delta_{j}^{\left(vj\right)} \end{array}\right., \end{array}$$

(3) $$\begin{array}{c} \Delta_{j}^{\left(vj\right)}＝\sum_{i＝1}^{m_{ij}^{\left(vj\right)}}D_{ij}^{\left(vj\right)}P_{ij}^{\left(vj\right)}. \end{array}$$

After entering the probability space *T*_*v*_ from the Euclidean space E, Δ_*j*_^(*vj*)^ is the distance error in the probability space from *w*_*j*_ to *v*_*j*_. In the formula 3, *D*_*ij*_^(*vj*)^ is the value of the Euclidean distance in the distance segment of the No. i (i = 1, 2,…., *m*_*ij*_^(*vj*)^) and *P*_*ij*_^(*vj*)^ is the probability distribution of the Euclidean distance *D*_*ij*_^(*vj*)^ after it enters the probability space Tv. This formula means the probability distribution of each segment is independent of the distance error. It accords with the fact that the distance scale in probability space has no symmetry [9]. Imitating the above method, it is obvious that we can calculate the distances from the point *r* in the Euclidean space to the point *v* in the probability space *T*_*v*_ and the point *w* in the probability space *T*_*w*_ separately.

**Figure 2:**
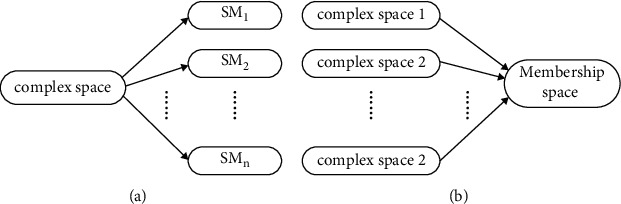
Two mapping methods of complex space *n*.

Figure 2 is the schematic diagram of two space-map methods of solving complex problems. As shown in Figure 2(a), mapping a complex problem to *n* spaces can be called the actinoid mapping [17]. For example, in order to extract and recognize the deeper information around a car, the environment image can be mapped into many mapping images by means of geometric and physical models. Figure 2(b) is the schematic diagram of mapping several spaces into one, and it is called the astriction mapping. For example, in the problem of multiobjective control, it is hard to find an optimal control point for many objective functions in different spaces. With the astriction mapping, it is possible to map the multiobjective functions into a membership fuzzy space so as to get the optimal control point in the unified form.

**Figure 3:**
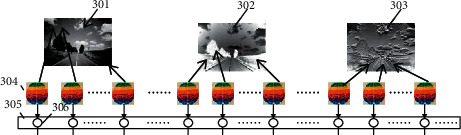
Actinoid mapping suitable for environment images of automatic vehicles.

Figure 3 is the schematic diagram of the actinoid mapping suitable for environment images of automatic vehicles. There are two key steps that will influence the accuracy of image recognition, information extraction, and pattern recognition.

As shown in Figure 3, 301 is the raw image around a car, while 302 and 303 are the mapping images of raw images separately. The deep information in the environmental images of cars can be extracted by applying the geometric and physical mapping models. 304 is obtained by the eigenvalues extracted from some areas of the mapping images through unsupervised learning. 305 is a perception layer in the neural network, and 306 is a node in this perception layer. In pattern recognition, the quality of information extraction is more important than the machine-learning classification algorithm. Based on the space mapping theory, the deep information in images surrounding cars and the high-order maximum probability of eigenvalues, which is able to represent the regional features and transcend the values derived from traditional statistics, can be extracted directly by mapping one image to many ones with geometric or physical models.

As shown in Figure 3, in order to extract more information from automobile's environmental images, it is necessary to make use of various spatial mapping for the environmental images and obtain more region segments to distill the features for each mapping image. In the model of Figure 3, the nodes of perception layers are able to be added infinitely so that it is possible to recognize the automobile images in the arbitrary depth, which will not introduce the extra complexity (only around *O*(*n*^2^)) and aggravate the difficulties of image recognition surrounding automobiles. Therefore, it is prospective that this model is more reasonable and capable of exceeding the traditional deep learning that increases the recognition effects by adding the number of hidden layer nodes.

**Figure 4:**
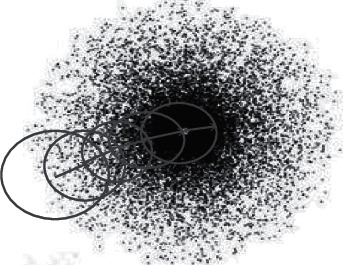
The schematic diagram of the machine learning model.

As shown in Figure 4, assuming there is a set of probability distributions belonging to a probability space in the Euclidean space, the number of elements in the set is *ζ*(*g*_*f*_ ∈ *G*,(*f* = 1,2,…, *ζ*)), and there must exist a characteristic value *A*(*G*) in *g*_*f*_ (*f* = 1, 2,…, *ζ*). Since the probability space is a measure space, a probability scale *M* [*G*, *A*(*G*)] must exist to satisfy the conditions below, and it can be taken as the reference to make iterations, which will make *G* migrate to the direction of the maximum probability.

(4) $$\begin{array}{c} A^{\left(n\right)}=A\left(G^{\left(n\right)}\right),\\ M^{\left(n\right)}=M\left[G^{\left(n\right)},A\left(G^{\left(n\right)}\right)\right],\\ G^{\left(n\right)}=G\left\{\left[A\left(G^{\left(n-1\right)}\right),M\left[G^{\left(n-1\right)},A\left(G^{\left(n-1\right)}\right)\right]\right]\right... \end{array}$$

*A*(*G*^(*n*)^) can be set as the characteristic value with maximum probability, and *M*[*G*^(*n*)^, *A*(*G*^(*n*)^)] can be the maximum probability scale whose center is *A*(*G*^(*n*)^). The unsupervised machine-learning model shown in Figure 4 is based on the theory of self-organizing based on a probability scale. It is because of the introduction of maximum probability scale *M*[*G*^(*n*)^, *A*(*G*^(*n*)^)] and the self-organizing calculation method taking *M*[*G*^(*n*)^, *A*(*G*^(*n*)^)] as a reference that the model of self-organizing based on a probability scale can get a breakthrough.

The maximum probability scale *M*[*G*^(*n*)^, *A*(*G*^(*n*)^)] is the probability scale containing the probability attributes of any one of normal distribution, multivariable normal distribution, logarithm normal distribution, exponential distribution, *t* distribution, *F* distribution, *X*^2^ distribution, binomial distribution, minus binomial distribution, multinomial distribution, Poisson distribution, Erlang distribution, hyper-geometric distribution, geometric distribution, traffic distribution, Weibull distribution, triangular distribution, beta distribution, gamma distribution.

The maximum probability scale *M* is also the scale about statistical properties or distances of probability spaces, including Mahalanobis distance, functions based on Gaussian processes, Wasserstein Distance scale, KL (Kullback–Leibler) Distance scale, P (Pearson) Distance scale, and probability measure of fuzzy event.

The maximum probability scale *M* can also be introduced as the Euclidean Distance scale, such as Manhattan Distance scale, Chebyshev Distance scale, Minkowski Distance scale, Cosine scale according to nonprobability space.

While acting as any one of Jaccard-similarity Coefficient scales, such as Hamming Distance scale or information entropy, it is another extended example for the maximum probability scale *M* to be used in the Bayesian analysis method to do self-organizing between the prior probability and the posterior probability.

The features of the unsupervised machine-learning model shown in Figure 4 are the little effect of the initial position, the migration to the maximal direction by itself, the spanning solution of high-order maximum probability over the traditional statistics, the directly obtainable probability distribution information of objective functions, the distributed machine-learning system.

## 4. The Implementation of Knowledge Acquisition with SDL in Automatic Driving

In this section, we will make a concrete introduction to the implementation of excellent drivers' knowledge acquisition with SDL.

**Figure 5:**
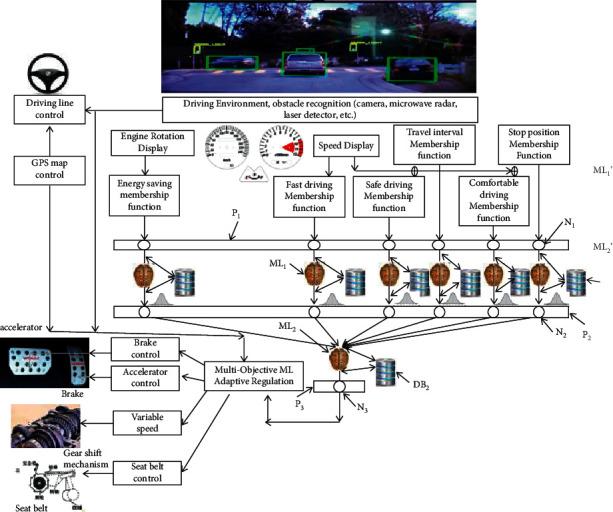
Machine-learning model of optimal multitarget control.

As shown in Figure 5, the problems of energy saving, safe driving, headway distance control, comfort driving are situated in the different spaces. There are three issues needing to be solved in the construction of the machine-learning model in the optimal multitarget control:

1. Mapping multitarget functions from the different spaces to the same one
2. Using machine learning to implement the knowledge acquisition of excellent drivers to imitate their driving skills
3. Regulating multitarget functions online automatically and rapidly by means of machine learning

We will mainly focus on issue 2 in this paper, while issue 1 and issue 3 will be emphatically discussed in another paper.

Based on the machine learning through excellent drivers' driving for many times, the values reflected by energy saving, safe driving, headway distance, comfort driving, quickly driving, and stopping distance will form a high-order maximum probability distribution function via the corresponding unsupervised machine learning ML_1_, which puts the data gotten from the knowledge acquisition into the database DB_1_.

There are many states for automatic vehicles:

- State 1: the car runs forward, and there are no barriers and other cars around it
- State 2: the car runs forward, and there is a car on the left behind
- State 3: the car runs forward, and there is a car on the left front
- State 4: the car runs forward, and there is a car on the left
- State 5: the car runs forward, and there is a car on the right behind
- State 6: the car runs forward, and there is a car on the right front
- State 7: the car runs forward, and there is a car on the right
- State 8: the car runs forward, and there is a car in the front
- State 9: the car runs forward, and there is a car on the back
- ……
- State *n*: ….

Each state corresponds to a different result acquired from multitarget control machine learning, so the driving state should be determined before the unsupervised machine learning, and every state should be corresponding to this state. In other words, the main purpose of multitarget optimal control machine learning in automatic driving is to achieve a probability distribution from the driving data of excellent drivers by many times machine learning based on a set of fuzzy values of multitarget optimal control in each driving state.

**Figure 6:**
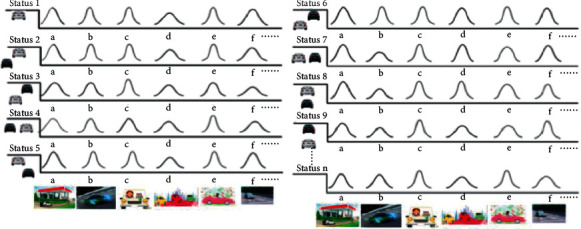
Knowledge acquisition of an excellent driver implemented by machine learning.

Figure 6 is a schematic diagram of knowledge acquisition of excellent drivers implemented by machine learning. As shown in Figure 6, in order to implement the knowledge acquisition, each probability distribution processed by machine learning, from state 1 to state *n*, corresponds to each result of multitarget optimal control machine learning. All the states, from (a) to (f), represent energy saving, safe driving, headway distance, comfort driving, quickly driving, and stopping distance separately. Actually, each of them reflects the corresponding probability distribution of each state. The greater the degree of probability dispersion is, the less the strict requirement of the objective function of this state is, and vice versa. The states of automatic driving are more than 9, and the method mentioned here can cover all the states, from 1 to *n*, and so is the objective functions.

In the normal process of automatic driving, a state can produce a set of objective function values, which will be sent to each node (N1) of the perception layer (P1) in Figure 5. The results of every objective function, which is the probability distributions obtained by processing the objective functions represented in Figure 6 from (a) to (f) through the machine learning (ML_1_) in Figure 5, will be used to calculate the distance from a node in the Euclidean space to the center of probability distribution in the probability space based on Figure 1, formula (1), which leaps over the Euclidean space to the probability space. At last, the final result will be sent to every node (N2) of the neural layer (P2) in Figure 5.

Based on the probability scale acquired by processing the final result with every node (N2) in the neural layer (P2) in Figure 5 through the self-organizing machine learning, it is possible for us to make a decision to adjust the objective function with the maximal probability scale. Meanwhile, it is necessary to introduce a threshold *ζ* adjusted by hand to estimate whether the distance of the machine learning result will exceed it or not. If the number of objective functions whose distances got from the machine learning (ML2) exceeds *ζ* is odd, adjustments will be applied to these functions. If it is even, the objective function with the maximal distance will be adjusted, and it is also acceptable to adjust an odd number of objective functions whose distances exceed the threshold at the same time by sending their useful information of them to the nodes (N3) in the neural layer (P3).

In order to fully control the braking mechanism, the accelerator mechanism, the speed control mechanism, and the safety belt, one of control methods, including linear-system control, nonlinear-system control, optimal control, stochastic control, adaptive control, fuzzy control, qualitative control, predictive control, real-time expert control, should be introduced into the adjustment information in the nodes (N3) of the neural layer (P3).

However, since the force of the braking mechanism is correlated to the speed of cars, the different speeds have to match the different forces of the braking mechanism. It is a waste of time to adjust the braking mechanism repeatedly in the self-organize way of the traditional methods, which makes the performance of emergency response inefficient. In order to make it meet the qualification, the parameters of self-organize control can be used as the input data in machine learning and then put them into the control system.

So is the accelerator mechanism. Every time using the data gotten by the machine learning based on the parameters that the accelerator mechanism needs for the self-organize control, the speed of control can be accelerated.

All the operations above are implemented in the center of self-organize control in the base of the multitarget machine learning. Each adjustment above needed to return to the machine learning (ML1, ML2) to judge whether it is valid or not. To get the same effect as excellent drivers can drive, it is necessary to carry out the circle of learning and adjusting for many times. The center of self-organize control based on the multitarget machine learning is also able to control the steering wheel according to the result of lane line recognition and route marked in GPS.

The environment recognition is also implemented by the dispersed machine learning. Figure 3 shows the process of mapping environment images to several mapping images, such as SM1 and SM, by using physical or geometrical models. It should also be considered to accord with the practical needs. For instance, in cognitive recognition, we need to map the lane line in environment images at first. And then, the mapping images will be divided into many regions, while every region corresponds to an unsupervised learning ML1. At last, the high-order maximum probability eigenvalues extracted from the corresponding region will be assigned to the node N1 in the perception layer P1.

The unsupervised learning ML2 between the node N1and the node N2 in the perception layer P1and P2 must have the capacity of obtaining the probability distribution from the same region of the same image by many times learning and storing it in the database DB1.

The unsupervised learning ML3 between the node N2 and the node N3 in the perception layer P2 and P3 has the function of recognizing images and is able to use environment images collected by cameras and data about the probability distribution from the DB1 to calculate the distance leaping over the Euclidean space to the probability space. Generally, the image recognition should accord to practical needs, just like lane line and burst image.

## 5. Conclusion

In this paper, we proposed a multitarget optimization control theory to learn and acquire the excellent drivers' knowledge in automatic driving system. The super deep learning (SDL) model we built based on this theory can execute the separate machine learning task for every part of automatic driving systems so that the optimization control of every segment in an automatic driving system would be possible. In order to perceive the environment around automobile even in the harsh climate, we introduced an image spatial mapping theory to extract the deep information of environmental images. Due to the complexity of the integral control of automatic driving, we proposed a knowledge acquisition theory based on skills of excellent drivers to deal with the fusion problem of multitarget optimal control, whose effect may exceed that of the fuzzy inference method used in automatic trains. Theories proposed in this paper still need to be experimented and confirmed by the concrete automatic driving platform, such as the self-driving car testing using search-based procedural content generation [18, 19].

## Data Availability

The data that support the findings of this study are available upon request to the corresponding author.
